# Pharmacokinetics/pharmacodynamics of glucocorticoids: modeling the glucocorticoid receptor dynamics and dose/response of commonly prescribed glucocorticoids

**DOI:** 10.5599/admet.2414

**Published:** 2024-10-19

**Authors:** David G. Levitt

**Affiliations:** Department of Integrative Biology and Physiology, University of Minnesota, Minneapolis, MN 55455, USA

**Keywords:** Dexamethasone, prednisone, prednisolone, methylprednisolone, cortisol, transcription

## Abstract

**Background and purpose:**

The main features of the dynamics of the glucocorticoid receptor (GR) have been known for 50 years: 1) in the absence of glucocorticoid (G), the receptor is localized entirely in the cytoplasm; 2) upon G binding, GR is converted into a tightly bound G form and is rapidly imported into the nucleus where it can bind DNA and modulate transcription; 3) nuclear export of GR is very slow; and 4) the nuclear form of GR can recycle through an unbound form, back to the bound transcription modulating form without leaving the nucleus.

**Experimental approach:**

A kinetic model that captures these features is presented, a set of model parameters for dexamethasone is derived, and the clinical implication for the commonly used glucocorticoids is discussed.

**Key results:**

At the high concentrations normally used to describe G pharmacodynamics, the model reduces to the standard Michaelis-Menten equation with a *K*_m_ that is a function of 4 model parameters. At very low concentrations, it reduces to another Michaelis-Menten equation with about a 1000-fold greater affinity, *eg.* at the nadir human endogenous cortisol concentration, the full model GR activity is 2.6 times greater than that predicted by extrapolation of the high concentration results.

**Conclusion:**

The model is used to relate normal human 24-hour endogenous plasma cortisol levels to transcriptional activity and is applied to the commonly prescribed glucocorticoids (dexamethasone, methylprednisolone, prednisone) in an attempt to provide a pharmacological rationale for the very large therapeutic dosage range that has been traditionally used.

## Introduction

Soon after the discovery of the glucocorticoid receptor (GR) in 1968 [1], Munck and colleagues [2,3] worked out the basic outlines of its mechanism. Upon binding of the glucocorticoid (G), the GR undergoes a series of conformational and locational changes driven by ATP-dependent phosphorylation in which the G becomes tightly bound deep inside the receptor and the receptor is actively transported into the nucleus where it binds to specific DNA glucocorticoid-response elements (GRE), modulating (either increasing or decreasing) transcription. Intensive investigations (and a massive literature) in the following years have fleshed out the details of the GR dynamics: the ATP-dependent phosphorylation events [4], the X-ray structure of GR with a completely enclosed tightly bound G [5], the role of chaperone modulation of GR [6,7], the details of the GR-DNA interactions and transcription regulation [8-10] and the kinetics of the various conformational and translocational steps (see below).

Glucocorticoids are some of the most commonly prescribed drugs [11]. They have an unusually high therapeutic window, ranging from low maintenance dosages of 5 mg/day to very large short-term dosages of 250 to 8000 mg/day prednisone equivalents [12,13]. The rationale for these dosages is based primarily on traditional regimens dating back 50 years or more [14], with little reference to the more recent research on glucocorticoid pharmacology. Considering the clinical importance of the glucocorticoid pharmacodynamic (PD) dose/response relationship, it is surprising that, seemingly, there has not been a previous attempt to develop a detailed PD model based on the known GR dynamics. The purpose of this paper is to develop such a model and describe its clinical implications.

The Experimental section outlines the kinetic model and the different GR states. The model is a function of 8 parameters: 2 equilibrium constants and 6 rate constants. The experimental measurements that constrain these parameters are described and an approximate quantitative set of values for dexamethasone (DEX) is provided. The results and discussion section presents a detailed description of the steady state and time-dependent properties of the model and the PD dose/response for the normal endogenous human daily plasma cortisol concentration. This normal basal transcriptional activity is then compared with that produced by the traditional dosages of the commonly prescribed glucuronides dexamethasone, methylprednisolone, and prednisone.

A Supplementary meterial containing two supplements that provide important background information. Supplement I describes the details of the derivation of the steady state and time-dependent GR model equations. Supplement II describes the details of the human pharmacokinetic (PK) models of dexamethasone, methylprednisolone and prednisone that are used in the main text to predict the free plasma concentrations following arbitrary intravenous (IV) or oral doses.

Although there are clearly some GR functions that are cytoplasmic [15,16], this analysis is focused on the great majority of GR that acts via DNA transcriptional modulation. In discussing the cytoplasmic versus nuclear location of GR, it will be assumed that all the cytoplasmic GR is potentially destined for nuclear import, whereas, in reality, there will be a small cytoplasmic fraction that is not associated with the nuclear import biochemistry.

## Experimental

### Glucocorticoid receptor kinetic model

**Figure 1.**
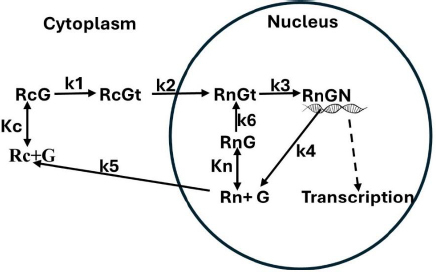
Glucocorticoid receptor kinetic model. The receptor cycles through 7 states: unbound in the cytoplasm (Rc) and the nucleus (Rn), a loose equilibrium G bound form in the cytoplasm (RcG) and nucleus (RnG), a tightly bound form in the cytoplasm (RcGt) and nucleus (RnGt), and the form that can bind DNA (RnGN), modifying transcription. The model is characterized by two equilibrium binding constants (Kc and Kn) and 6 rate constants (k1 to k6)

The kinetic model is shown in Figure 1. The receptor has 7 different states: unbound in the cytoplasm (Rc) and the nucleus (Rn), a loose equilibrium G bound form in the cytoplasm (RcG) and nucleus (RnG), a tightly bound form in the cytoplasm (RcGt) and nucleus (RnGt), and the form that can bind DNA (RnGN), modifying transcription. The model approximates the reaction pathway and conformational states described by Pratt *et al.* [7]. It is a simplified version of the actual glucocorticoid receptor dynamics because the multiple chaperones and cofactors that are bound to the receptor in the different states are not included. Each reaction in Figure 1 presumably consists of a series of reactions as the cofactors are assembled or disassembled. It is hoped that the model in Figure 1 captures the essential features of the kinetics. The single-headed arrows represent irreversible unidirectional transitions driven either by ATP phosphorylation and/or chaperone-driven conformational changes. The initial binding of G (characterized by equilibrium constants *K*c and *K*n) drives GR into a tightly bound conformation (at rate constants *k*1 and *k*6) in which the G is buried deep in the protein [5]. The nuclear import and export (characterized by the rate constants *k*2 and *k*5) are complex energy-dependent processes involving dynein, the nuclear pore complex and a variety of chaperones and accessory proteins [6]. The only states in which there is reversible G binding is Rc ⇔ RcG and Rn *⇔* RnG. For all the other states, the G is buried deep inside the protein and cannot exchange. The nuclear form of the receptor (RnGt) is converted to the form RnGN (at rate constant *k*3), which can potentially interact with DNA and modulate transcription. For a complete model description, one must also specify the total amount of receptor (*R*tot). The following analysis is primarily focused on, *e.g.* the concentration dependence of the transcriptional rate relative to the maximum rate at very high G concentration. For this case, *R*tot is just a scaling factor that can simply be set to 1.

A crucial aspect of the model is that the receptor can recycle from the active form (RnGN), through the free nuclear form Rn (rate constant *k*4) back to the active form (rate constants *k*6 and *k*3) without leaving the nucleus [17-19]. Since most of the receptor is in the nucleus even at low glucocorticoid concentrations, this implies that the active RnGN state is determined primarily by the nuclear receptor affinity (*K*n), not the cytoplasmic affinity (*K*c).

### Estimates of model parameters

This section describes an estimate of the model parameter values (Table 1) for DEX, the commonly used glucuronide in GR mechanistic studies. The rate constants for nuclear import (*k*2) and export (*k*5) were measured in older studies by following the movement of tritiated labeled glucocorticoid bound to receptor [20] and, more recently, by imaging of green fluorescent protein fused GR (GFP-GR) [6,21-23] or immunofluorescence [19,24]. The rate of nuclear import is fast, with a time constant of about 5 min (*k*2 = 0.2 min^-1^), while the rate of export is very slow, with a time constant of about 9.8 hours (*k*5 = 0.0017 min^-1^). This dramatic difference in the rate of import and export is a characteristic of GR kinetics and plays a critical role in determining the G concentration dependence of transcriptional modulation.

**Table 1.** Model parameters for dexamethasone and cortisol

Parameter	Dexamethasone	Cortisol
*R*_tot_	1	1
*K*c / nM	5	75
*K*n / nM	155	1550
*k*1 / min^-1^	10	10
*k*2 / min^-1^	0.2	0.2
*k*3 / min^-1^	0.2	0.2
*k*4 / min^-1^	0.04	0.04
*k*5 / min^-1^	0.0017	0.0017
*k*6 / min^-1^	1.0	1.0

Although the rates of conversion of the loosely bound to the tight cytoplasmic form (*k*1) has not been experimentally measured, one would expect it to be fast relative to the nuclear import rate (*k*2) that it is in series with and, therefore, not rate limiting. A time constant of 0.1 min (*k*1 = 10 min^-1^) has been assumed. As shown below, *k*1 is the most important parameter in determining the apparent affinity of the system at very low concentrations. Although the diagram in Figure 1 suggests that *k*6 and *k*1 should have a similar rate, this is misleading, and *k*6 will be slower than *k*1. This is because the process involved in nuclear import (= *k*2) involves a number of cofactor additions necessary to convert RcGt to the RnGt state, and these steps must also be involved in *k*6, which has been assumed to have a value of 1 min^-1^. These estimates of *k*1 and *k*6 are only rough guesses.

Another parameter that can be directly measured, although under limiting conditions, is *K*c. In the absence of glucocorticoid, all receptors are in the cytoplasmic state *R*c. Because the conversion to the tight form RcGt is an active ATP-driven process, *k*1 = 0 for measurements at 0 °C, and GR binding measurements should directly measure *K*c. The dexamethasone (DEX) affinity (≈ *K*c) of homogenized cells at 0 °C is about 1 nM [25,26]. The extrapolation of this result to 37 °C is uncertain, and a value of *K*c = 5 nM has been assumed. As discussed below, the value of *Kc* only becomes important for GR transcriptional regulation at very low concentrations. Since there are no accurate measurements in this concentration range, there is no direct experimental evidence for the value of *K*c at 37 °C.

The rate that the nuclear form of the receptor recycles through the unbound form and back to the tightly bound form without leaving the nucleus is determined by the rate constants *k*6, *k*3 and *k*4 (Figure 1). These rates can be estimated from the approximately 25-minute half-time of the [^3^H]DEX dissociation rate from nuclear receptors when a 200-fold excess of unlabeled DEX is added [17,27]. It has been assumed that the slowest rate in this recycling is *k*4, which has been assigned a value of *k*4 = 0.04 min^-1^ (time constant = 25 min) and that *k*3 *= k*2 = 0.2 min^-1^.

The most important experimental GR-related measurement is the steady state G concentration dependence of the transcriptional rate. Recently, detailed results for a variety of glucocorticoids have been obtained based on measurements of GR-mediated activation of a transfected reporter gene in a well-defined cell line [28-32]. The experimental concentration dependence is usually well described by the standard, simple Michaelis-Menten relation Equation (1) where *V*maxT and *K*mT indicate the *V*max and *K*m experimental constants determined from transcriptional activation measurements:



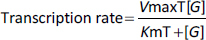

(1)


where [G] is the free plasma glucocorticoid concentration. The GR concentration dependence is characterized by the parameter *K*mT, which is equal to the commonly measured *EC*_50_ (or, for the case where G inhibits transcription, *IC*_50_). The *K*mT for DEX varies for different cell lines, ranging from 1 to 10 nM [28-32], and a *K*mT of 5 nM has been assumed.

## Results and discussion

### Steady-state glucocorticoid receptor model expressions

Based on the model in Figure 1, it will be assumed that the transcriptional rate is proportional to the fraction of the GR receptor that is in the RnGN state. The steady-state expression Equation (2) for the dependence of RnGN on the glucocorticoid concentration G is a complicated function of all the model parameters (see Supplement I):



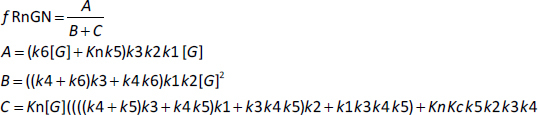

(2)


where *f*RnGN is the fraction of the receptor that is in the RnGn state (=RnGn*/R*tot). As discussed above, a characteristic of GR kinetics is the relatively rapid rate of nuclear import (5 min) versus an extremely slow rate of export (10 hours). This means that, except at very low G concentrations, the receptor is almost entirely nuclear. Thus, for the range of concentrations that are normally experimentally measured, Equation (2) can be simplified by assuming that the receptor is 100 % nuclear. For this case, remarkably, Equation (2) reduces to the Michaelis-Menten form Equation (3) (see Supplement I) where *V*maxH and *K*mH are the *V*max and *K*m determined in the high concentration limit:



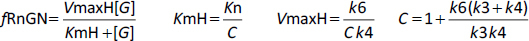

(3)


As expected, the kinetics in this limit does not depend on any of the cytoplasmic model parameters (*K*c, *k*1, *k*2, *k*5). Assuming that *K*mH for DEX is equal to the experimentally measured *K*mT (Equation (1)) of 5 nM, and using the assumed values of k6, k3, and k4 (Table 1) in Equation 3, C = 31, *V*maxH = 0.769, and *K*n = 155 nM (Table 1). This value of *K*n is just an estimate because it depends on the assumed values of *k*3, *k*4 and *k*6, values that, as discussed above, are only rough guesses. There are no direct experimental measurements of *K*n.

The kinetic model (Figure 1) exhibits interesting behavior in the very low G concentration limit where the nuclear equilibrium binding of G to R_n_ is negligible and there is no nuclear recycling of the receptor. In this limit, Equation (2) again reduces to a Michaelis-Menten form Equation 4 (see Supplement I) where *V*maxL and *K*mL are the *V*max and *K*m determined in the low concentration limit:



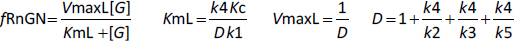

(4)


Substituting the assumed values of *k*2, *k*3, *k*4, *k*5 and *K*c (Table 1) in Equation (4), *D* = 31.5, *V*maxL= 0.0317 and *K*mL = 0.000787 nM. Thus, at very low concentrations (less than about 1 % of *K*mT), the system behaves as if it has an extremely high G affinity of about 0.0008 nM, 6 thousand times greater than the experimental affinity measured at high concentrations (*K*mT *= K*mH) of 5 nM! Again, this is only a rough estimate because it depends on the estimated values of *k*1, *k*2, *k*3, *k*4, *k*5 and *K*c. This apparent high affinity arises from the peculiar features of the GR dynamics, with the relatively fast nuclear import and slow nuclear export of GR. The *V*maxL (=0.0317) of this high-affinity limit is also very low, only about 4% of the high concentration *V*maxH (=0.769) and, thus, can only produce about 4% of the maximum transcription rate. This result indicates that even at G concentrations normally considered negligible (*e.g.*, DEX concentration of 0.001 nM), there should still be a residual transcriptional activation rate of about 4 % of the maximum rate.

The value of *D* in Equation (4) is dominated by the *k*4/*k*5 term, so that, to a first approximation, they reduce to Equation (5):



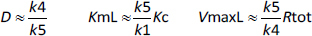

(5)


As discussed above, although *k*5 can be directly measured, there are no experimental measurements of *k*1 and its assumed value of 10 min^-1^ in Table 1 was just a guess. Thus, the apparent *K*m at very low concentrations (= *K*mL), which is inversely proportional to *k*1 is highly uncertain. However, the conclusion that at low concentrations, there is very high affinity is relatively robust because even if *k*1 is only 1 min^-1^ (the absolute limit allowed by the experimental results), ten times slower than assumed in Table 1, *K*mL =0.0079 nM, still an extremely high affinity. In contrast, the parameters that determine *V*maxL are reasonably well constrained by the time-dependent experiments, and the above estimate is probably accurate within a factor of 2.

### Steady-state model predictions for dexamethasone and cortisol

**Figure 2.**
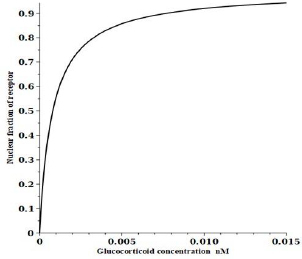
Fraction of receptor that is located in the nucleus as a function of glucocorticoid concentration using the dexamethasone parameters in Table 1

Using the above estimated model parameter set (Table 1), the quantitative properties of the model will be discussed in this section. Figure 2 shows a plot of the steady state fraction of the receptor that is in the nucleus (= Rn + RnG+RnGt+RnGN) as a function of the DEX concentration. A DEX concentration of only about 0.01 nM is enough to shift 90 % of the receptor to the nucleus.

**Figure 3.**
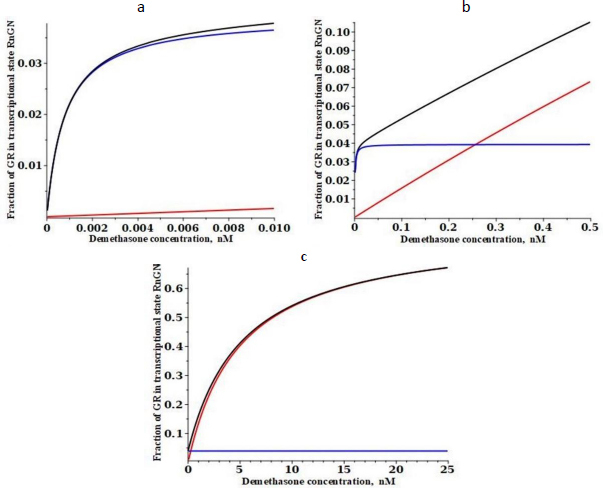
Steady state glucocorticoid concentration dependence of the fraction of receptor that is in the state RnGN (see Figure 1) that can potentially modify the transcription rate for the high-affinity dexamethasone (Table 1) with its apparent experimental transcriptional *K*mT of 5 nM. Three different concentration ranges are shown: very low (a), intermediate (b) and high concentration (c). The black line is the exact solution (Equation 2), the red line is the high concentration approximation (Equation 3) and the blue line is the low concentration approximation (Equation 4)

Figure 3 shows a plot of the DEX concentration dependence of the steady-state fraction of the GR that is in the RnGN state that can potentially modulate transcription. The black line is the exact solution (Equation (2)), the red line is the high-concentration approximation (Equation (3)) and the blue line is the low-concentration approximation (Equation (4)). The top panel shows the predicted extremely high apparent affinity (0.0008 nM) predicted by Equation (4) in the low concentration limit. Similarly, the bottom panel shows that a Michaelis-Menten equation with the apparent *K*mH*= K*mT of 5 nM predicted by Equation (4) provides a good fit to the high concentration range over which experimental PD measurements are usually made.

**Figure 4.**
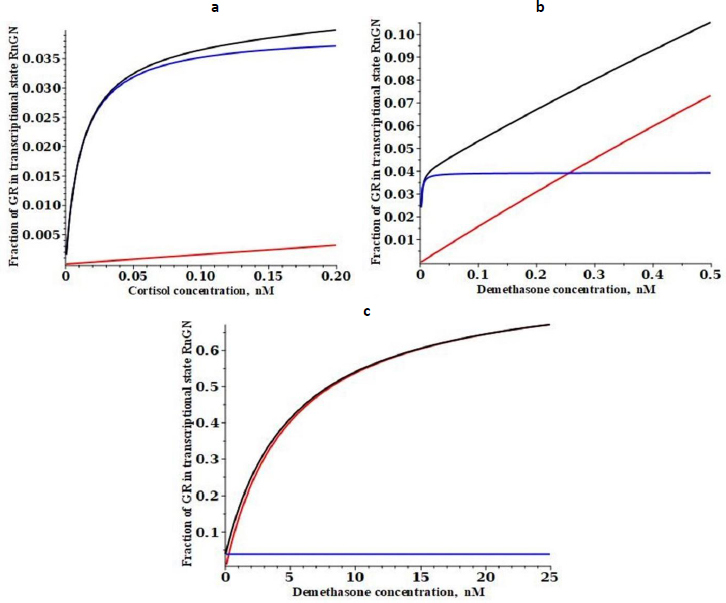
Steady state glucocorticoid concentration (nM) dependence of the fraction of receptor that is in the state RnGN (see Figure 1) that can potentially modify the transcription rate for the low-affinity cortisol (Table 1) with its apparent experimental transcriptional *K*mT of 50 nM. Three different concentration ranges are shown: very low (a), intermediate (b) and high (c). The black line is the exact solution (Equation 2), the red line is the high concentration approximation (Equation 3) and the blue line is the low concentration approximation (Equation 4)

Figure 4 shows a similar plot for the low-affinity cortisol. It is assumed that the only parameters that are G-dependent are the two binding constants (*K*c and *K*n) and that the rate constants are not G-dependent (see Table 1). Cortisol has a fifteen-fold lower affinity than DEX for the cytoplasmic receptor at 0 °C (15 nM for cortisol versus 1.1 nM for DEX)[26]. Making the same assumption that the 37 °C affinity is fivefold less, the cortisol affinity *K*c is assumed to be 75 nM. The experimental *K*mT for transcriptional modulation by cortisol ranges from 35 to 67 nM for various cell lines [30,31], and it will be assumed that it is 50 nM, 10 times that of DEX. From Equation (3), this corresponds to a cortisol *K*n of 1550 nM (Table 1).

### Time-dependent model predictions for dexamethasone

**Figure 5.**
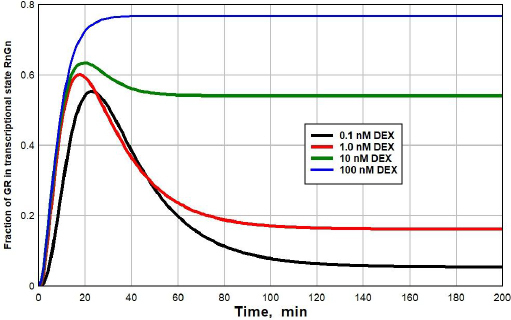
Time course of the fraction of GR in the transcriptional modifying state RnGN as a function of time after the addition of varying concentrations of DEX

Figure 5 shows a plot of the time dependence (see Supplement I for details) of the fraction of GR that is in the RnGN state after the DEX concentration at time = 0 is suddenly raised from 0 to 0.1 nM (black line), 1 nM (red), 10 nM (green) or 100 nM (blue). At the low concentrations (0.1 or 1 nM), there is a transient spike in the RnGN fraction as the GR cycles through RnGN (see Figure 1) before it dissociates at rate = *k*4 (time constant = 25 minutes) to the free nuclear receptor. After about 100 minutes, the fraction of RnGN settles down to the steady-state values described in Figure 3. Although this transient spike is a novel prediction of the kinetic model, it probably is not clinically significant because the time course of the transcriptional modulation of protein synthesis is so slow (five or more hours [33,34]) that these short-term spikes should be averaged out.

**Figure 6.**
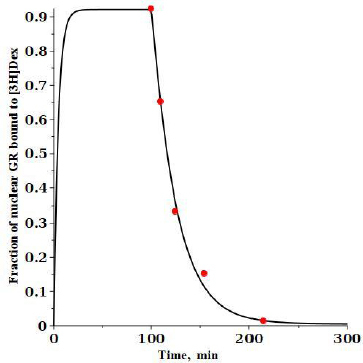
Time course of labeled [^3^H]dexamethasone tightly bound to nuclear GR receptor following a cold chase with a 200-fold excess of unlabeled dexamethasone. At time 0, 100 nM [^3^H]dexamethasone is added. At time of 100 minutes, 20 mM unlabeled dexamethasone is added. The fraction of [^3^H]dexamethasone labeled nuclear receptor (RnG*t + RnG*N) is plotted as a function of time. The solid circles are the experimental data of Meijsing *et. al.* [17]

An experimental check of the model predictions is provided by the DEX dissociation measurements of Meijsing *et al.* [17]. Cells were equilibrated with 100 nM [^3^H]DEX and then the time course of hormone dissociation from GR was measured following a cold chase with the addition of a 200-fold excess of unlabeled DEX. Figure 6 shows the model predictions for these conditions (see Supplement I for details).

At time 0, 100 nM of labeled DEX (G*) is added and then, after a steady state is reached at 100 minutes, 20 mM of unlabeled DEX is added, and the fraction of the labeled DEX tightly bound to nuclear GR receptor (= RnG*t + RnG*N) is plotted. The model predictions are in good agreement with the experimental results of Meijsing *et al.* (red solid circles) [17].

### The glucocorticoid pharmacodynamics of endogenous cortisol and varying doses of dexamethasone, methylprednisolone and prednisone

The GR nuclear transcriptional rate will first be estimated for the normal human endogenous diurnal 24-hour free plasma cortisol concentration. This basal activity will then serve as a reference for the common clinically used glucocorticoids. At high plasma G concentrations, the details of the above GR model can be ignored and one can simply use the experimental steady state Michaelis-Menton relation (Equation (1)) whose affinity is characterized by the parameter *K*mT *(= K*mH*)* which is equal to the experimental *EC50* (or *IC50* for an inhibitory effect) of the GR cellular response. However, this equation is not valid at low concentrations where, as discussed above, the affinity may increase more than 1000-fold and the exact model result (Equation (2) must be used. This is quantitatively illustrated in the following discussion.

The best defined *EC50* measurements are from experiments in which a well-defined cultured cell line is transfected with a specific GLE reporter gene (*e.g.*, luciferase), and the rate of production of the transcription product is measured (with an allowance for a delayed response) as a function of the free (unbound) glucocorticoid concentration in the cell culture medium. There have been many such measurements for cortisol, DEX, MP and prednisolone, summarized in Table 2. The second column in Table 2 lists the absolute value of the DEX *K*mT, while the *K*mT for the other glucocorticoids are expressed relative to that of dexamethasone. Each row corresponds to a different cell line. For comparison, the last row (Goodman and Gillman [35]) lists the approximate relative potency for the human clinical response.

**Table 2.** Cell culture *K*_mT_ values for selected glucocorticoids

Cell line	*K*mT / nM (Dexamethasone)	*K*mT (relative to dexamethasone)			Ref.
		Cortisol	Methylprednisolone	Prednisolone	
AZ-GR	9.55	6.91	3.16	4.67	[30]
HeLa	12	11	3.33	3.33	[36]
A549	10	15	4	7	
HTC	4	15	5	5	
GR-LBD	0.154	30.9	2.87		[37]
PBMC	4.1		3.36	21	[38]
CCL-202	0.5	20		10	[39]
GR/3xGRE	1.66	27.5			[40]
GR/MMTV	1.02	58.9			
GR-LBD	0.83	72.4			
Clone #1	2.82			5.37	[28]
Clone #5	5.89			4.79	
Clone #6	8.71			3.98	
A549	1	10			
Hepatocytes	10				[31]
GR(WT)	0.5				
GR(ER22)	8				
AR42	10				
Human clinical response		25	5	6.25	[35]

Considering that these clinical results include pharmacokinetic factors such as oral absorption, metabolic rates, volume of distribution, etc., they are surprisingly similar to the cell culture results. For a given glucocorticoid, there is as much as a 10-fold variation of *K*mT, depending on the cell line. This suggests that it is not appropriate to assume a single value of *K*mT for the human glucocorticoid response which, presumably, involves a number of different cell types. A “representative” set of *K*mT values was assumed (Table 3), with the understanding that these values are only rough estimates. The uncertainty in the predicted model concentration dependence is roughly proportional to this variation in *K*mT.

**Table 3.** Assumed representative glucocorticoid model affinity parameters

	*K*mH = *K*mT / nM	*K*n / nM	*K*c / nM	*K*mL /nM
Dexamethasone	5	155	5	0.00079
Cortisol	50	1550	75	0.0118
Prednisolone	15	465	15	0.0044
Methylprednisolone	15	465	15	0.00236

There are two situations where the exact GR model may become important and the simple experimental Michaelis-Menten Equation (1) is not valid. The first is at very low G concentrations where, as discussed above, the apparent affinity may be increased by a factor of 1000 or more. The second situation is the question of the validity of the steady state Michaelis-Menton relation in the presence of time-varying plasma G concentration. The GR cycles through a series of states with some time delays (see Figure 5), and the steady state assumption may not be accurate. Both of these situations are discussed below. For the calculations that use the complete model, it will be assumed that the rate constants *k*1 *– k*6 are G independent (Table 1) and only the equilibrium constants *K*n and *K*c are glucocorticoid dependent. The value of *K*n in Table 3 is determined from Equation (3) with *K*mH *= K*mT. The values of *Kc* for DEX and cortisol were discussed above (Table 1) and it was assumed that *K*c *= K*mH for MP and prednisone. In all the following model plots, to eliminate initial value effects, the calculations are run for two days and only the second day is plotted.

**Figure 7.**
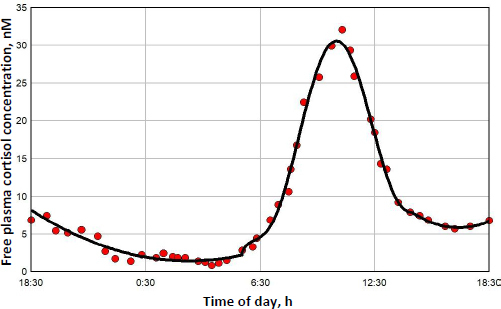
Daily endogenous human free plasma cortisol

The diurnal endogenous cortisol calculations are based on the measurements of Bhake *et. al.* [41] of the human daily free unbound cortisol concentrations. They used microdialysis to obtain nearly continuous values of the free subcutaneous cortisol, which, it will be assumed, should be nearly equal to the free plasma concentration. There is considerable individual variation in the results, and Figure 7 shows a representative 24-hour measurement. The red circles are the individual measurements, and the solid line is the piecewise polynomial fit to the data that is used in the following calculations of the transcriptional activity.

**Figure 8.**
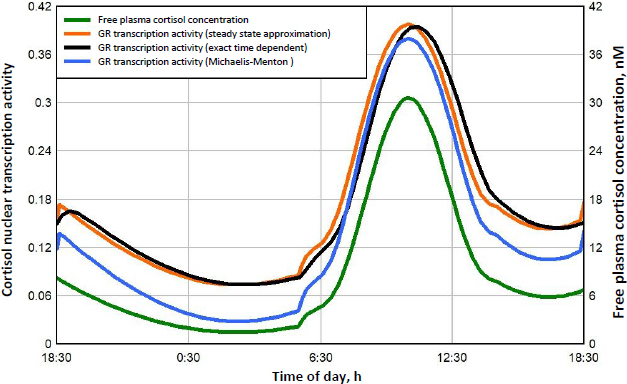
Endogenous human cortisol nuclear transcription activity as fraction of maximum possible. The free plasma cortisol concentration (green line) is indicated on right Y axis, and the GR transcriptional activity for three different approximations (red, black and blue lines) is indicated on left Y axis

Figure 8 shows this daily variation in free plasma cortisol (green line) and the corresponding transcriptional activity for three different model assumptions. Because the nuclear GR continually cycles through a series of states (Figure 1), not all the receptors can be in the RnGN state, even at infinite glucocorticoid concentration (see Figure 5). In Figure 8, the left Y coordinate is the value fraction of the GR that is in the state RnGN relative to the maximum fraction of GR in RnGN *(= RnGNmax* = 0.806) for an infinite cortisol concentration. The blue line is the simple high concentration Michaelis-Menton relation (Equation (1) using the experimental *K*mT *= K*mH. The red line is the exact model steady state expression (Equation (2)) assuming that the nuclear activity is proportional to the fraction of GR in the RnGN state. The black line is the exact RnGN fraction determined from solving the set of time dependent differential equations that describe the model (see Supplement I for details). To eliminate initial value effects, the model results were determined for 2 days, and the plotted data is for the second day.

As expected, although the experimental Michaelis-Menton relation (blue line) is a good approximation at high concentrations, it significantly underestimates the activity at low concentrations. For example, at the nadir cortisol concentration of 1.45 nM at about 3 AM, the exact time dependent activity (black line) is 0.074, 2.6 times greater than the Michaelis-Menton activity (blue line) of 0.028. The steady state model result (red line) is nearly identical to the exact time dependent result (black line), with only a small-time delay in the activity. In the following calculations for DEX, MP and prednisone, only the exact steady state model result (Equation (2)) will be used for the estimation of GR transcriptional activity.

A new quantitative measure of the glucocorticoid potency is introduced: the “glucocorticoid transcription quotient” (GTQ) which is defined as the integral of the GR activity (as a fraction of the maximum possible for infinite G dose) averaged over 24 hours:



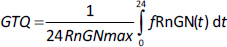

(6)


where *f*RnGN(*t*) is the fraction of GR in the RnGN state as a function of time and *RnGNmax* is the maximum fraction for an infinite glucocorticoid dose. *GTQ* = 1 for an infinite dose. For endogenous cortisol (Figure 8), the *GTQ* is 0.17. Table 4 summarizes the *GTQ* for cortisol and for varying doses of DEX, MP and prednisone.

**Table 4.** Glucocorticoid transcription quotient (GTQ). (q.d. = one dose every 24 h, b.i.d. = one dose every 12 h)

Glucocorticoid	Dose	GTQ
Cortisol	Endogenous	0.17
Dexamethasone	1 mg IV q.d.	0.29
	10 mg IV q.d.	0.736
	5 mg IV b.i.d.	0.788
	40 mg IV q.d.	0.91
	1 mg oral q.d.	0.29
	10 mg oral q.d.	0.75
	40 mg oral q.d.	0.91
Methylprednisolone	5 mg IV q.d.	0.19
	20 mg IV q.d	0.35
	10 mg IV b.i.d	0.45
	80 mg IV q.d.	0.54
	40 mg IV b.i.d.	0.72
	320 mg IV q.d.	0.73
	160 mg IV b.i.d.	0.90
Prednisone	5 mg oral q.d.	0.24
	10 mg oral q.d.	0.31
	5 mg oral b.i.d.	0.41
	20 mg oral q.d.	0.40
	100 mg oral q.d.	0.60
	50 mg oral b.i.d.	0.81
	400 mg oral q. d.	0.77

The determination of the transcriptional activity of DEX, MP and prednisone is obtained by substituting the free plasma concentration as a function of time [G] into the steady state model relation Equation (2). The PK that is used to predict the free plasma concentration following different oral and IV dosages is described in detail in the attached file Supplement II. The activity is expressed as the fractional activity relative to the maximum possible. To eliminate initial value effects, the dose is given for two days, and the second day results are used.

Dexamethasone (DEX) has the highest GR affinity (Table 1) and is the longest lasting (slowest clearance, see Supplement II), both of which contribute to making it the most potent of the commonly used glucocorticoids. As discussed above, it is the glucocorticoid of choice for most *in vitro* experiments. It is administered either orally as DEX or IV as DEX-phosphate which is rapidly converted to DEX. DEX has simple linear PK with a free plasma fraction of about 0.23. It is usually administered as short-term therapy, often as a “pulse”, at periodic intervals. The dosages range from a low of about 1 mg/day, to “high doses” of 40 mg/day. The PK determination of the plasma concentration following arbitrary IV or oral doses is described in detail in the attached file Supplement II.

**Figure 9.**
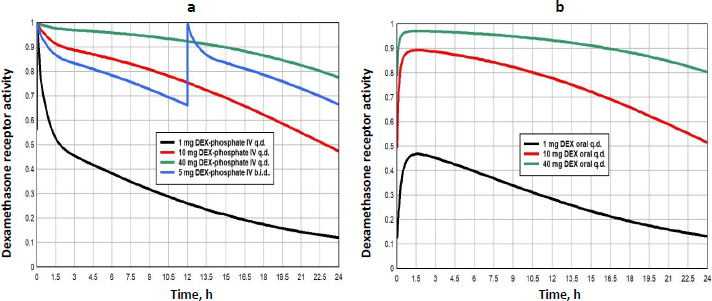
DEX GR activity following a -3 q.d. and b.i.d. and b – 3 oral g.d. doses per day

Figure 9 shows the GR transcriptional activity of varying dosage regimens of either IV DEX-phosphate (left panel) or oral DEX (right panel). The *GTQ* of the different doses are listed in Table 4. The *GTQ* of the “low” 1 mg dose is 0.29, 70 % greater than that of endogenous cortisol. The “high” single dose of 40 mg (either IV or oral) nearly maximizes the activity for a 24-hour period with a *GTQ* of 0.91. Although 1 mg DEX-phosphate (MW 472.4) is equivalent to 83 % of a 1 mg oral DEX (MW 392.5), because the oral DEX bioavailability is only 59 %, the IV dose represents a 40% greater systemic dose. Despite this, the *GTQ* of the IV and oral doses are nearly identical because the slow oral DEX absorption spreads out the plasma DEX over a longer period.

Methylprednisolone (MP) is a widely used glucocorticoid, administered either orally or intravenously (IV) as the acetate (Depo-Medrol) or succinate (Solu-Medrol) derivative. It has simple linear pharmacokinetics over a large range of dosages and non-saturable plasma binding with a free fraction of about 0.23. The clinical methylprednisolone doses range from low dosages of 20 mg/day[42], to high doses of 1000 mg/day or higher, usually as short term “pulse” doses [12,43,44]. The detailed PK modeling that is used to predict the free concentration following arbitrary oral or IV doses is described in the attached file Supplement II.

**Figure 10.**
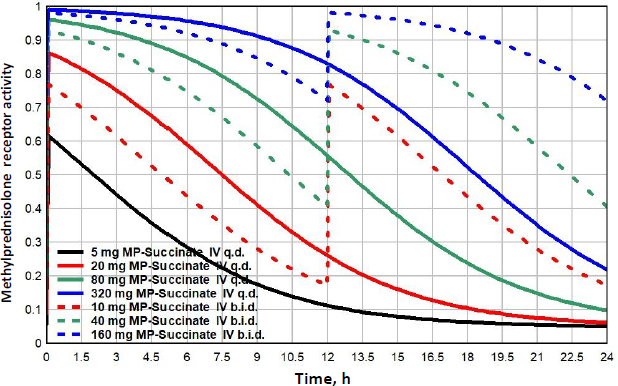
Methylprednisolone GR activity

Figure 10 shows the MP GR transcriptional activity for a total daily IV dosage of 5 mg (black), 20 mg (red), 80 mg (green) or 320 mg (blue), given as once/day (q.d., solid lines), or twice/day (b.i.d., dashed lines). Table 3 lists the corresponding *GTQ*. The 5 mg q.d. dose has a *GTQ* of 0.19, 12 % greater than endogenous cortisol. Because of the relatively short MP lifetime, even the highest dose (320 mg/day q.d., solid blue line) has a *GTQ* of only 0.73 and does not fully activate GR. Dividing this 320 mg dose into two 160 mg doses at 12-hour intervals (b.i.d, dashed blue line.) increases *GTQ* to 0.90.

Prednisone, which is only administered orally, is the most commonly prescribed glucocorticoid [11] which is unfortunate because, unlike DEX and MP, it has complicated non-linear PK which adds uncertainty to its modeling [45-47]. It is a prodrug that is converted to the active prednisolone by the liver when it is absorbed. Prednisolone has a high affinity binding to the plasma protein transcortin (also known as corticosteroid-binding globulin (CBG)), which has a limited binding capacity that saturates at high prednisolone concentrations. This means that, as the concentration increases, the unbound free fraction increases, increasing both the clearance and the volume of distribution. Further complicating the kinetics, the plasma prednisone and prednisolone are continually interconverting, and the plasma prednisolone/prednisone ratio varies non-linearly over a range of 2.7 to 10, depending on the prednisolone concentration. Xu et al. [47] have developed a kinetic model that provides a good prediction of the time course of the free plasma prednisolone concentration ([G] in Equation 2) following a large range of oral prednisone doses. The corresponding GR receptor activity is obtained by substituting this value of G into Equation (2). The non-linear prednisone PK and its modeling is discussed in detail in the attached file Supplement II.

**Figure 11.**
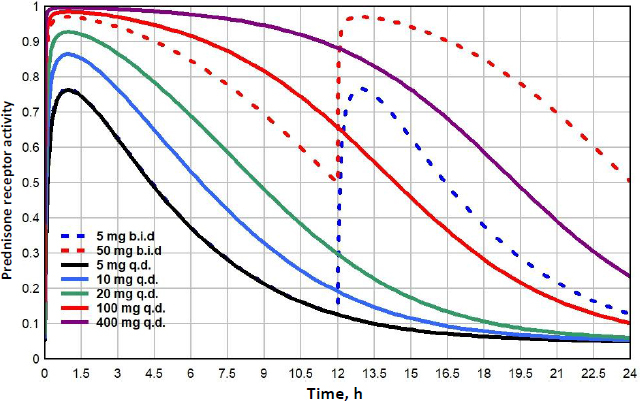
Prednisone GR activity

Prednisone doses range from lows of 5 to 10 mg/day, medium doses of about 20 mg/day, high doses of 100 mg/day to very high pulse doses to 250 mg/day or greater [48]. Figure 11 shows the GR receptor activity following oral doses ranging from 5 to 400 mg/day. Because the low prednisone doses are often administered chronically for long periods, it is of particular interest to compare their action with that of endogenous cortisol. Although the 5mg/day dose (black line) is usually considered “physiological”, it has a *GTQ* of 0.24, 41% greater than that of endogenous cortisol. In addition, it has a significantly different time course, rising to a peak activation of 0.76, nearly twice the peak of 0.39 for endogenous cortisol (Figure 8). Because of its relatively rapid clearance, even a 400 mg q.d dose only has a *GTQ* of 0.77. Dividing the dose into two b.i.d. doses significantly increases the activity. For example, 100 mg given as two 50 mg doses (red dashed line) has a greater *GTQ* than 400 mg q.d.

## Conclusion

Although the main qualitative features of the glucocorticoid receptor (GR) dynamics have been understood for nearly 50 years, there has not been a previous attempt to quantitively model the GR dynamics. The kinetic model described in Figure 1 is, seemingly, the minimal one that incorporates all the essential features. Unquestionably, it is an oversimplification of the true kinetics, but it is hoped that it captures the main features. Because the model parameters are only roughly constrained by experimental data, the quantitative results described above should only be regarded as first approximations. It is hoped that this model may stimulate more detailed investigations of these parameters.

In the presence of moderate G concentrations, GR is predominantly recycling in the nucleus without reentering the cytoplasm (Figure1). In this concentration range, the activity is described by the standard Michaelis-Menten Equation (3) with a *K*m equal to the *K*mT determined experimentally from the concentration dependence of transcriptional modulation. One implication of this is that, over most of the operational G concentration range, the GR transcriptional activity *K*mT should be independent of the affinity of the cytosolic free receptor *K*c. This is supported by the results of Mercier *et. al.*[25] who reported that two derived hepatoma cell lines had about a 7-fold difference in *K*mT, despite having identical *K*c (measured at 0 °C). The *K*mT varies by a factor of as much as 10 for different cell line systems (Table 2). It is usually assumed that this variation is a result of variations in the binding affinities *K*c or *K*n. However, *K*mT (= *K*mH) depends not only on *K*n, but also on the three rate constants *k*3, *k*4 and *k*6 (Equation (3)) and variations in any of these 4 parameters could account for the cell line differences.

A novel result of the kinetic analysis is that, at very low G concentrations, the GR activity is described by the Michaelis-Menten Equation (4) with an extremely high apparent affinity *K*mL (Table 3). This low concentration range is physiologically important for estimates of the transcriptional activity of human endogenous cortisol. As shown in Figure 8, at the nadir cortisol concentration, the model predicted transcriptional activity is 2.7 times greater than what would be predicted by extrapolation of the experimental high concentration Michaelis-Menten relation (blue line). This low concentration range is also important for estimates of the transcriptional activities of low concentrations of methylprednisolone or prednisone where, as can be seen in Figs. 10 and 11, the activity does not fall to zero at long times but, instead, levels off at about 0.04 (4 % of the maximum activity) because of this high affinity.

There are, at least, two rather simple experiments suggested by this analysis that could confirm (or refute) the model predictions. The first is a test of the prediction of the very high affinity of GR at very low G concentration. These studies would have to be able to detect a residual activity of about 4% of the maximum at, *e.g.*, a DEX concentration of 0.01 nM or less. The second is a test of the model prediction of the dependence of the high concentration *K*mH *(=K*mT) on the model parameters *K*n, *k*3, *k*4 and *k*6 (Equation (3)), Specifically, the intrinsic *K*n of the unbound nuclear receptor should be about 30 times greater than the experimentally measured *K*mT, (Table 3). It should be possible to directly measure *K*n by, first, incubating cells at 37 °C in DEX for 30 minutes, so that all receptor is nuclear, then, switching to a DEX free medium for about 30 minutes, so that the nuclear receptor goes into the unbound Rn state (but still is nearly all nuclear) and then, at 0 °C, measure the receptor binding constant *K*n in lysed cells.

Most of the GR mechanistic studies have used DEX as the test glucuronide and the rate constants *k*1 *– k*6 listed in Table 1 are based on these studies. In applying the model to the lower affinity cortisol, methylprednisolone and prednisone, it was assumed that only the equilibrium binding constants *K*c and *K*n depend on G, while the rate constants *k*1 *– k*6 are G independent. This might be expected because, in the tightly bound states, the G is buried deep inside the protein so that the protein surface with its chaperone and auxiliary binding sites should not depend on G. However, Schaaf et. al. [49] have reported that the pattern of nuclear binding of GR-yellow fluorescent protein is definitely G dependent, suggesting that some of the rate constants might also be G dependent.

Glucocorticoids are used clinically over a huge dosage range. The rational for these dosages, especially in the higher dosage range, is based primarily on traditional regimens dating back 50 years or more [14]. This lack of a rigorous pharmacological foundation is in part explained by the complicated pharmacodynamic (PD) dose-response relationships of glucocorticoids [50]. Not only is the GR involved in the complex dynamics discussed here (Figure 1), but, since the result of the DNA transcriptional modification may take 6 hours or more to reach maximum effect and may last for many days, it is difficult to quantify the dose-response. The *GTQ* (Equation (6)) is introduced here as an approximate cell culture based approach to quantifying the PD. It assumes that the G activity is determined by the daily average of the fraction of the GR that is in the conformation that can potentially bind to and modify DNA. The *GTQ* for endogenous cortisol and varying doses of DEX, MP and prednisone are summarized in Table 4. Although it is obviously a great oversimplification, neglecting factors such as GR turnover and downregulation [51,52] and cytosolic GR effects [15,16], it is hoped that the it may provide a useful approach to quantifying the PD of glucocorticoids.

Supplementary material Additional data are available at https://pub.iapchem.org/ojs/index.php/admet/article/view/2414, or from the corresponding author on request.
