# Supplementary material for: Pharmacokinetics/pharmacodynamics of glucocorticoids: modeling the glucocorticoid receptor dynamics and dose/response of commonly prescribed glucocorticoids
Source: ADMET DMPK. 2024 Oct 19;12(6):971–89. doi: 10.5599/admet.2414 (PMC11661806; doi:10.5599/admet.2414)
Supplement: Supplementary file 1 — Supplementary material [file ADMET-12-2414-S1.pdf]

ADMET & DMPK 12(6) (2024) S25-S34

Open Access : ISSN : 1848-7718

http://www.pub.iapchem.org/ojs/index.php/admet/index

Supplementary material to

Pharmacokinetics/pharmacodynamics of glucocorticoids: modeling the glucocorticoid receptor dynamics and dose/response of commonly prescribed glucocorticoids

David G. Levitt

Department of Integrative Biology and Physiology, University of Minnesota, Minneapolis, MN 55455, USA

ADMET & DMPK **12(9)** (2024) 971-989; https://doi.org/10.5599/admet.2414

Supplement I: Derivation of glucocorticoid receptor model steady state and time dependent equations

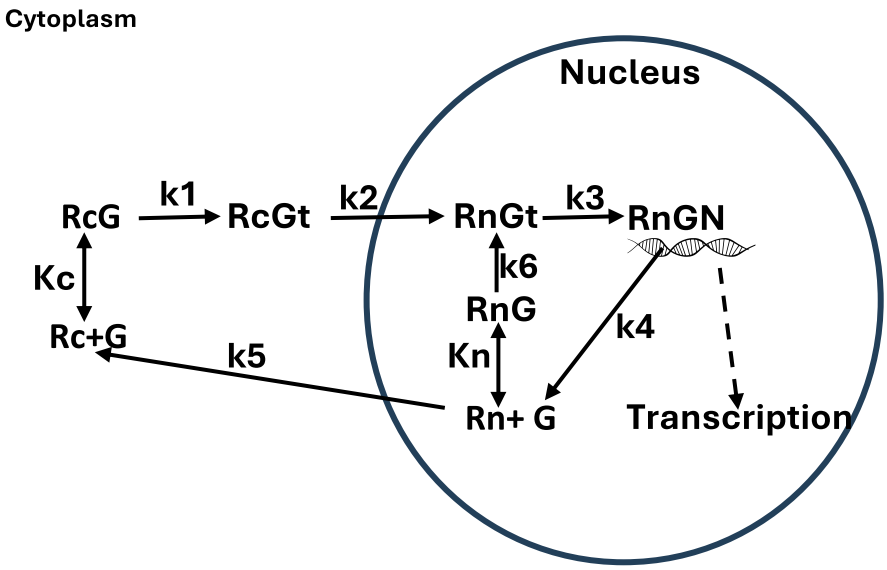

General steady state solutions

Define *Tc* and *Tn* as the total amount in the Rc-RcG and Rn-RnG equilibrium pair with equilibrium constants *Kc* and *Kn*, respectively ([G] = glucocorticoid concentration):

$Kc = Rc[G]/RcG \quad Kn = Rn[G]/RnG \quad Tc = Rc+RcG \quad Tn = Rn + RnG$

Express *Rc*, *RcG*, *Rn* and *RnG* in terms of *Tc* and *Tn*:

$$Rc = \frac{Tc\,Kc}{Kc+[G]} \qquad RcG = \frac{Tc[G]}{Kc+[G]} \qquad Rn = \frac{Tn\,Kn}{Kn+[G]} \qquad RnG = \frac{Tn[G]}{Kn+[G]} \tag{1}$$

Steady state for *Tc*:

$$k5\,Rn = k1\,RcG \Rightarrow \frac{k5\,Tn\,Kn}{Kn+G} = \frac{k1\,Tc\,G}{Kc+G}$$

$$Tn = A\,Tc \qquad A = \frac{k1\left(Kn+G\right)G}{k5\,Kn\,(Kc+G)} \tag{2}$$

Using steady state conditions for RcGt, RnGt and RnGN, express each of these states in terms of *T*c and *T*n:

$$\mathrm{RcGt} = \frac{k1[G]T\mathrm{c}}{k2(K\mathrm{c}+[G])} \tag{3}$$

$$RnGt = \frac{k1[G]T\mathrm{c}}{k3(K\mathrm{c}+[G])} + \frac{k6T\mathrm{n}[G]}{k3(K\mathrm{n}+[G])} \tag{4}$$

$$RnGN = \frac{k1T\mathrm{c}[G]}{k4\left(K\mathrm{c}+[G]\right)} + \frac{k6T\mathrm{n}[G]}{k4(K\mathrm{n}+[G])} \tag{5}$$

Finally: Rtot = Tc + Tn +RcGt +RnGt +RnGN

Substituting the above expression for *RcGt, RnGt* and RnGN into the *R*tot expression:

$$T\mathrm{c} = \frac{R\mathrm{tot}}{B} \qquad B = 1 + A + \frac{k1[G]}{K\mathrm{c}+[G]}\left(\frac{1}{k2}+\frac{1}{k3}+\frac{1}{k4}\right) + \frac{Ak6[G]}{K\mathrm{n}+[G]}\left(\frac{1}{k4}+\frac{1}{k3}\right) \tag{6}$$

This completes the steady state solution since all the above expressions for the receptor states are in terms of *T*c (or *T*n *= A T*c), and *T*c is described by Equation 6.

Steady state solution in limit of high concentration where it can be assumed all receptor states are in the nucleus.

This limit of the steady state is simple because there is no branching:

*k*4 RnGN = *k*6 RnG = *k*6 Tn[G]/(*K*n+[G]) = k3 RnGt

$$RnGt = \frac{k6\ T\mathrm{n}\ [G]}{k3(K\mathrm{n}+[G])} \qquad RnGN = \frac{k6\ T\mathrm{n}\ [G]}{k4(K\mathrm{n}+[G])} \tag{7}$$

Substituting these two expressions in *R*tot = *T*n+*RnGt*+*RnGN* and solving for *RnGN*:

$$RnGN = \frac{V_{\max}[G]}{K\mathrm{m}+[G]} \qquad V_{\max} = \frac{k6R\mathrm{tot}}{k4\ C} \qquad K\mathrm{m} = \frac{K\mathrm{n}}{C} \qquad C = 1 + \frac{k6(k3+k4)}{k3\ k4} \tag{8}$$

Steady state solution in limit of low concentration where it can be assumed that nuclear recycling is negligible:

Steady state for each receptor state*:*

k4 *RnGN = k*3 *RnGt = k*2 *RcGt = k*1 *RcG = k*5 Rn
*T*c = RcG (*K*c +G)/G ≈ RcG Kc/G = *k*4 RnGN *K*c/(*k*1 G)
*R*tot = *T*c + RcGt + RnGt +RnGN + *R*n

Express *T*c*, RcGt, RnGt* and *R*n in terms of *RnGN,* substitute in *R*tot equation, and solve for *RnGN*:

$$RnGN = R\mathrm{tot}\ [G] / \left\lfloor [G]\left(1 + k4/k3 + k4/k2 + k4/k5\right) + k4\ K\mathrm{c}/k1 \right\rfloor \tag{9}$$

This can also be written in Michaelis-Menten form:

$$RnGN = \frac{V_{\max}[G]}{K\mathrm{m}+[G]} \qquad V_{\max} = \frac{R\mathrm{tot}}{D} \qquad K\mathrm{m} = \frac{k4K\mathrm{c}}{Dk1} \qquad D = 1 + \frac{k4}{k3} + \frac{k4}{k2} + \frac{k4}{k5} \tag{10}$$

Time dependent solution for two competing identical glucocorticoids.

For the cold chase experiment where [^{3}H]dexamethasone is dissociated by the addition of a 200 fold excess of unlabeled dexamethasone, it is necessary to find the time dependent binding of the two competing forms (labeled =A and unlabeled =E) to the different receptor states.

Define: Tc = Rc + RcE + RcA Tn = Rn +RnE + RnA

*R*c, *RcE* and *RcA* are in instantaneous equilibrium, expressed in terms of *T*c:

RcE = Rc×E/Kc = Tc E/(Kc+E+G)
RcA = Rc×*A/Kc = Tc A/(Kc+E+A)

CC BY

R= Tc Kc/(Kc+E+A)

Similarly, *Rn, RnE* and *RnA* are in equilibrium, expressed in terms of *Tn*:

Rn = Tn Kn/(Kn+E+A) RnE = Tn E/(Kn+E+A) RnA = Tn A/(Kn+E+A)

The system is then defined by the 8 differential equations for the 8 states *Tc, Tn, RcEt, RnEt, RnEN, RcAt, RnAt* and *RnAN*:

d Tc/dt == k5 Rn - k1 RcE -k1 RcA = k5 Tn Kn/(Kn+E+A) - k1 Tc(E+A)/(Kc + E+A)
d RcEt/dt = k1 RcE – k2 RcEt = k1 Tc E/(Kc + E+A) - k2 RcEt
d RnEt/dt = k2 RcEt +k6 RnE – k3 RnEt = k2 RcEt + k6 Tn E/(Kn+E+A)- k3 RnEt
d RnEN/dt = k3 RnEt – k4 RnEN
d RcAt/dt = k1 RcA – k2 RcAt = k1 Tc A/(Kc + E+A) - k2 RcAt
d RnAt/dt = k2 RcAt +k6 RnA – k3 RnAt = k2 RcAt + k6 Tn A/(Kn+E+A) - k3 RnAt
d RnAN/dt = k3 RnAt – k4 RnAN
d Tn/dt = k4 RnEN +k4RnAN - k5 Rn – k6 RnE -k6 RnA = = k4 RnEN+k4 RnAn – k5 Kn Tn/(Kn+E+A) – k6 Tn(E+A)/(Kn+E+A)

This system of first order differential equations was then solved for arbitrary time dependent inputs of the concentrations of the two glucocorticoids (E and A) using Maple (Maplesoft).

Supplement II: Pharmacokinetics (PK) of dexamethasone (DEX), methylprednisolone (MP) and prednisone.

This supplemental file describes the derivation of the detailed PK equations that determine the free plasma glucocorticoid concentration following an arbitrary IV or oral dose. These concentrations are used in the main text to predict the glucocorticoid receptor nuclear transcription activity.

Linear pharmacokinetics: dexamethasone and methylprednisolone.

Most drugs have linear PK (prednisone is an important exception). That is, *e.g.* if one doubles the dose, the response is also doubled. This is an essential requirement for the application of the standard PK analysis. For linear kinetics, the plasma concentration is completely characterized by the "unit response function" $h(t)$, which describes the plasma concentration following a unit bolus input. The plasma concentration $C(t)$ for an arbitrary input $I(t)$ is then described by [1]:

$$C(t) = \int_0^t I(\tau)\, h(t-\tau)\, d\tau \tag{11}$$

The usual approach is to approximate $h(t)$ by a sum of exponentials, with the number of exponentials equivalent to the number of well-stirred interacting compartments:

$$h(t) = \sum_{i=1}^{N} a_i\, e^{-t/T_i} \tag{12}$$

Given the plasma concentration following an arbitrary IV input $I_{IV}(t)$, $h(t)$ can be determined from equation (11) by deconvolution.

Using this $h(t)$ and equation (11), one can then predict the plasma concentration following an oral dose if one knows the rate that the oral dose enters the systemic circulation (= $I_{GI}(t)$). I have previously described the use of this approach to characterize the intestinal absorption of a large series of solutes using the PK software PKQuest [2]. The time course of the intestinal absorption $I_{GI}(t)$ is well described by:

$$I_{GI}(t) = \frac{F\,D}{T_G - T_P}\left[e^{-t/T_G} - e^{-t/T_P}\right] \tag{13}$$

where D is the oral dose, F is the bioavailability (fraction of dose reaching the systemic circulation), T_G is the time constant for gastric emptying and T_P is the time constant for intestinal absorption, which is a measure of intestinal permeability. Given the plasma concentrations following a known IV and oral dose, PKQuest returns the optimal values of the $h(t)$ parameters (a_i, T_i) and the 3 parameters (F, T_G, and T_P) describing the intestinal absorption.

The PK of a linear system is characterized by two parameters: the steady state clearance (Cl_{ss} / l min^{-1}) and the volume of distribution (V_{ss} / l). They are defined for a system where there is a constant systemic input I_{ss} and the plasma concentration has reached a steady state C_{ss}:

$$Cl_{ss} = I_{ss} / C_{ss} \qquad V_{ss} = Amt / C_{ss} \tag{14}$$

where Amt is the total amount in the body. For a one-compartment system, the concentration $C(t)$ following a bolus input dose D is given by:

$$C(t) = \frac{D}{V_{ss}} e^{-(Cl_{ss}/V_{ss})t} \tag{15}$$

It can be shown that, for an arbitrary linear systems under rather general conditions, Cl_{ss} and V_{ss} can be determined from the plasma concentration $C(t)$ following a dose D given as an arbitrary input I(t):[1]

$$Cl_{ss} = D / AUC$$
$$V_{ss} = D[AUMC / AUC^2 - MIT / \mathrm{AUC}] \tag{16}$$
$$\mathrm{AUC} = \int_0^\infty C(t)dt \qquad AUMC = \int_0^\infty t\,C(t)dt \qquad MIT = (1/D)\int_0^\infty t\,I(\mathrm{t})dt$$

Table S1 lists the Cl_{ss} and V_{ss} determined using the $C(t)$ data from figs. 1S and 2S along with the free (unbound) fraction in plasma for dexamethasone and methylprednisolone.

Table S1. Linear PK parameters

Glucocorticoid	Cl_{ss} / L (min 70 kg)$^{-1}$	V_{SS} / L (70 kg)$^{-1}$	Free fraction
Dexamethasone	0.12	48.3	0.23
Methylprednisolone	0.415	98.3	0.23

The PK of DEX is linear[3-5]. It is about 77 % bound to albumin.[6,7] Since the plasma albumin concentration is about 40 g l^{-1}, or 600 µM, it would require a dose of more than 800 mg of DEX to saturate this binding. Thus, the albumin binding is not concentration dependent, which is one reason that DEX PK is linear. Because of its low solubility, intravenous DEX is administered as the phosphate derivative which, upon entering the blood, is rapidly converted to DEX [8,9]. This complicates the PK analysis because one needs to know the exact rate of this hydrolysis in order to determine the DEX IV input function, which is a requirement for the determination of $h(t)$. However, for DEX, the conversion is fast enough that one can approximate the DEX input by setting it equal to the DEX phosphate input.[4,8,10]

Figure S1 shows the experimental plasma concentration data (red circles) of O'Sullivan *et. al.* [10] following a 1 mg IV dose of DEX phosphate (Figure S1a), equivalent to 0.83 mg DEX, and 1 mg oral dose of DEX (Figure S1b) and the corresponding PK model fits (solid lines). It is necessary to use a 3-compartment model to accurately fit the data and the best fit $h(t)$ (equation (12)) and oral absorption (equation (13)) parameters are listed in Table S2. The bioavailability (F) for the averaged data shown in Fig. S1 is 0.59. There is a surprisingly large individual variation in the bioavailability, varying from 0.34 to 0.89 [10]. This probably results from the relatively low intestinal permeability (T_P = 222 min) which leads to varying fractions of DEX passing unabsorbed into the large intestine, depending on the small intestinal transit times.

(cc) BY

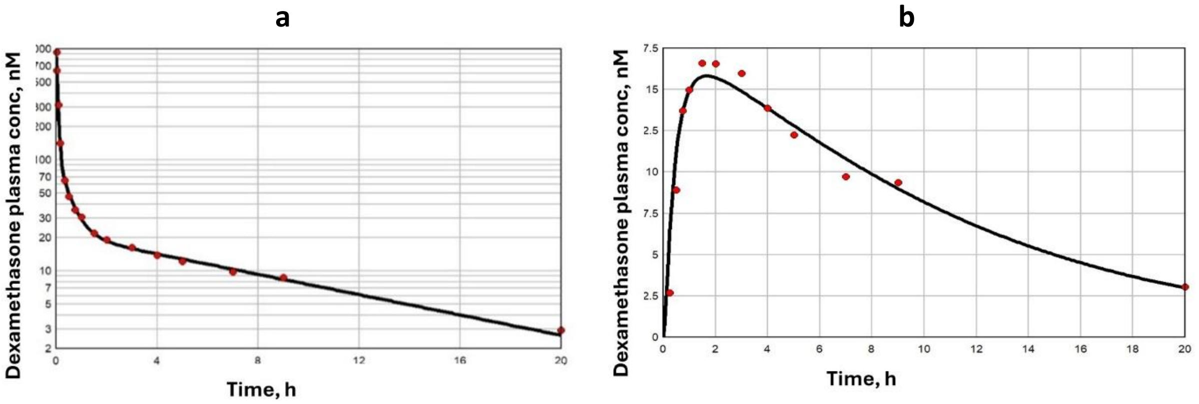

Figure S1. 3-compartment model fits to the experimental dexamethasone data of O'sullivan *et. al.* [10] following a 1 mg DEX phosphate (0.83 mg DEX) IV input (a) and a 1 mg DEX oral input (b). The solid lines are the model fits to the data using the unit input *h*(*t*) and oral input parameters listed in Table S2

Table 2S. Parameters characterizing the linear PK: N is the number of compartments required for the best fit, a_i and T_i are the *h*(*t*) parameters, and *F*, T_G and T_P characterize the intestinal absorption rate.

Glucocorticoid	*N*	a_1 / nM	a_2 / nM	a_3 / nM	T_1 / h	T_2 / h	T_3 / h	*F*	T_G / min	T_P / min
Dexamethasone	3	0.422	0.04	0.01	0.056	0.45	9.49	0.59	14	222
Methylprednisolone	1	0.0102			3.94			0.82	21	101

Methylprednisolone (MP) is 77 % bound to albumin [11] and also has linear PK [12, 13]. It is administered IV as the acetate, succinate or phosphate derivative which is rapidly converted to the free MP [12]. The most commonly used IV form is MP succinate (Solu-Medrol®). Figure 2S shows the model fit (solid line) to the experimental data of Groenewoud et. al. for a 100 mg IV input (left panel) of MP (as MP succinate) and a 100 mg oral MP input (right panel) using the 1-compartment *h*(*t*) and oral absorption parameters listed in Table S2. The MP data can be fitted with a simple 1-compartment model because it does not have the initial spike in plasma concentration seen for DEX (Fig. S1). This may indicate that the rate of hydrolysis of MP succinate is relatively slower than that of DEX phosphate.

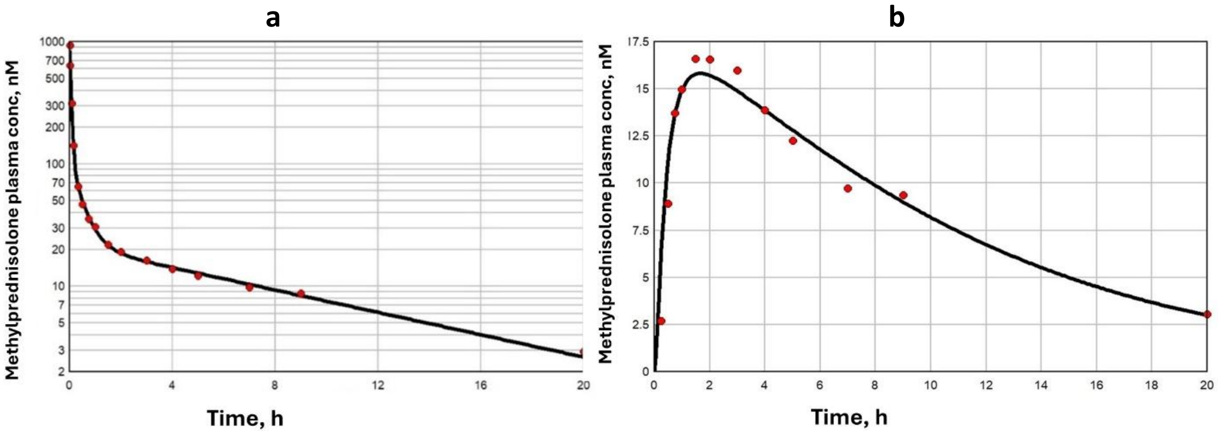

Figure S2. 1-compartment model fits to the experimental methylprednisolone data of Groenewoud et. al. following a 100 mg IV (a) and oral input (b). The solid lines are the model fits to the data using the unit input *h*(*t*) and oral input parameters listed in Table S2

Non-linear pharmacokinetics: Prednisone

Prednisone, the most commonly prescribed oral glucocorticoid, has very complicated kinetics[14-16]. It is a prodrug that is converted to the active prednisolone form by the liver when it is absorbed. Prednisolone has a high affinity binding to the plasma protein transcortin (also known as corticosteroid-binding globulin (CBG)). Transcortin has a limited binding capacity and saturates at high prednisolone concentrations. This

means that, as the concentration increases, the unbound free fraction increases. Only the unbound free fraction is metabolized and is distributed into the intracellular space. Since this free fraction increases as the dose increases, the corresponding clearance and volume of distribution will also increase non-linearly. Further complicating the kinetics, the prednisone and prednisolone are continually interconverting in plasma, and the plasma prednisolone/prednisone ratio varies non-linearly over a range of 2.7 to 10, depending on the prednisolone concentration. In addition to the non-linear transcortin binding complicating the PK, it is the free concentration that is important for the pharmacodynamic action discussed in the main text, and this will also by non-linearly related to the total concentration.

The magnitude of this non-linearity is illustrated in Fig. S3 which shows the Rose *et. al.* [15] plasma prednisolone concentration as a function of time after oral doses of 5 (black), 20 (red) and 50 (green) mg prednisone. The solid lines are the experimental results, and the dashed lines are the predicted plasma concentration using the 5 mg dose and assuming linearity. The linear prediction for the 50 mg dose is about twice the observed experimental result.

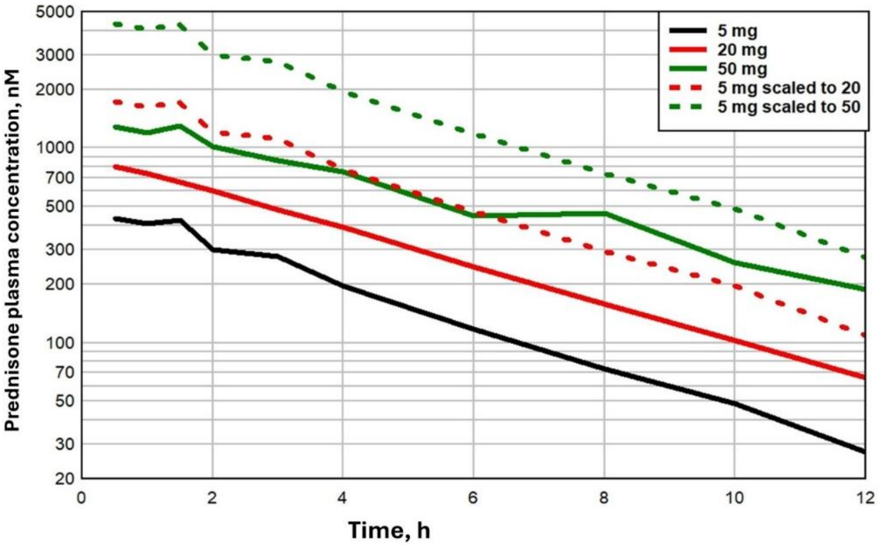

Figure S3. Plasma prednisolone concentration (nM) as a function of time following an oral prednisone dose of 5 (black), 20 (red) or 50 mg (green). The solid lines are the experimental results. The dashed lines show the predicted concentrations for the 20 and 50 mg doses based on the 5 mg dose and assuming the PK are linear

This non-linearity is primarily the result of the saturating high affinity transcortin prednisolone binding. An approximate quantitative analysis of this binding is given here. The transcortin binding is described by:

$$T + C \xrightleftharpoons{K_T} TC \quad \rightarrow \quad K_T = \frac{TC}{T \times C} \rightarrow \quad C = \frac{TC}{K_T \times T} \tag{17}$$

where *C* and *T* are the unbound prednisolone and transcortin concentrations, respectively, *TC* is the transcortin bound concentration, and K_T is the prednisolone-transcortin dissociation constant. Prednisolone also has a low affinity, non-saturating binding to albumin:

$$A + C \xrightleftharpoons{K_A} AC \quad \rightarrow \quad K_A = \frac{AC}{A \times C} \rightarrow C = \frac{AC}{K_A \times A} \tag{18}$$

Since the unbound plasma albumin concentration (*A*) is about 600 µM, it is not saturated by prednisolone and can be assumed to be a constant and equation (18) can be approximated by:

$$C = \frac{AC}{KA} \qquad KA = K_A A \tag{19}$$

where *KA* is a constant. If there is no transcortin binding (as is the case for DEX and MP):

(cc) BY

$$C = \frac{AC}{KA} = \frac{C_{tot} - C}{KA} \quad \Rightarrow \quad \text{Fractionunbound} = \frac{C}{C_{tot}} = \frac{1}{1+KA} \tag{20}$$

In addition, there are two equations for the total prednisolone (C_{tot}) and transcortin (T_{tot}) concentration:

$$C_{tot} = C + TC + AC \tag{21}$$

$$T_{tot} = T + TC \tag{22}$$

Solving the 4 equations (Equations (17), (18), (21) and (22)) for the 4 unknowns (C, TC, AC,T) for the free prednisolone concentration C:

$$C = \frac{B + (C_{tot} - T_{tot})K_T - KA - 1}{2K_T(KA+1)} \qquad B = \sqrt{(C_{tot} - T_{tot})^2 K_T^{\,2} + 2K_T(C_{tot} + T_{tot})(KA+1) + (KA+1)^2} \tag{23}$$

The three binding parameters (K_T, T_{tot}, KA) can be estimated from the experimental measurements of the free prednisolone (C) as a function of the total prednisolone (C_{tot}). Figure S4 shows a plot of predicted free percent (100 C/C_{tot}) as a function of C_{tot} compared to the experimental measurements of Rose *et. al.* [15] using the parameters T_{tot} = 500 nM, K_T = 0.02 (nM)$^{-1}$ and KA = 1. The free percent varies from about 8 % at low concentrations when the high affinity transcortin binding dominates, to 50 % at high concentrations when only the low affinity albumin binding is important.

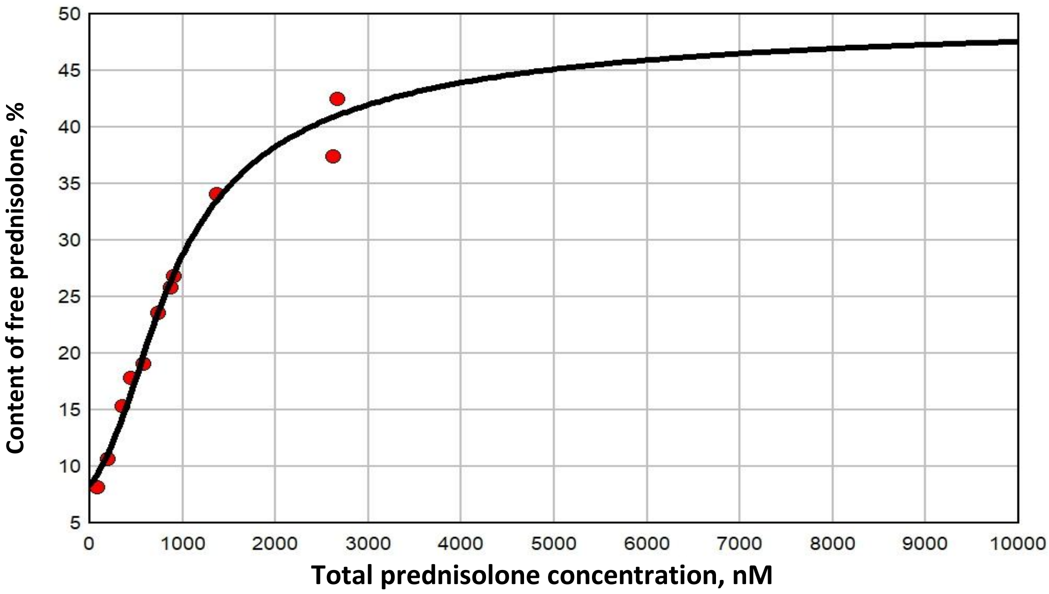

Figure S4. Content of free prednisolone as a function of the total prednisolone concentration. The experimental results of Rose *et. al* (red circles) are compared with the model predictions (solid line)

Xu *et. al.* [16] have developed an interesting model of the non-linear prednisone/prednisolone PK. They assume that the free prednisolone and prednisone concentrations can be described by a simple linear 1-compartment model, with the only non-linearity arising from the transcortin binding that relates the free to the total prednisolone. They derive a set of rate constants describing the rate of oral prednisone absorption, and rates of clearance and interconversion of free prednisone and free prednisolone. This model was used here to predict the free plasma prednisolone following oral prednisone doses of 5, 20 and 50 mg. The free prednisolone (C) was converted to the corresponding total prednisolone (C_{tot}) using the inverse of the binding equation (23):

$$C_{tot} = \frac{(KAK_T\,C + K_T T_{tot} + K_T\,C + KA + 1)C}{K_T\,C + 1} \tag{24}$$

Figure S5 compares this predicted total plasma prednisolone with the experimental results of Rose *et. al.* [15]. The 8 Xu *et. al.* [16] model parameters for the oral prednisone dose were used unchanged except that the rate constant for intestinal prednisone absorption was increased from 1.08 to 1.8 h^{-1}. The agreement between experiment and model over this 10-fold dose range is quite impressive considering the simplicity of the model and that the Rose *et. al.* data was not the primary data set that was used for the parameter determination.

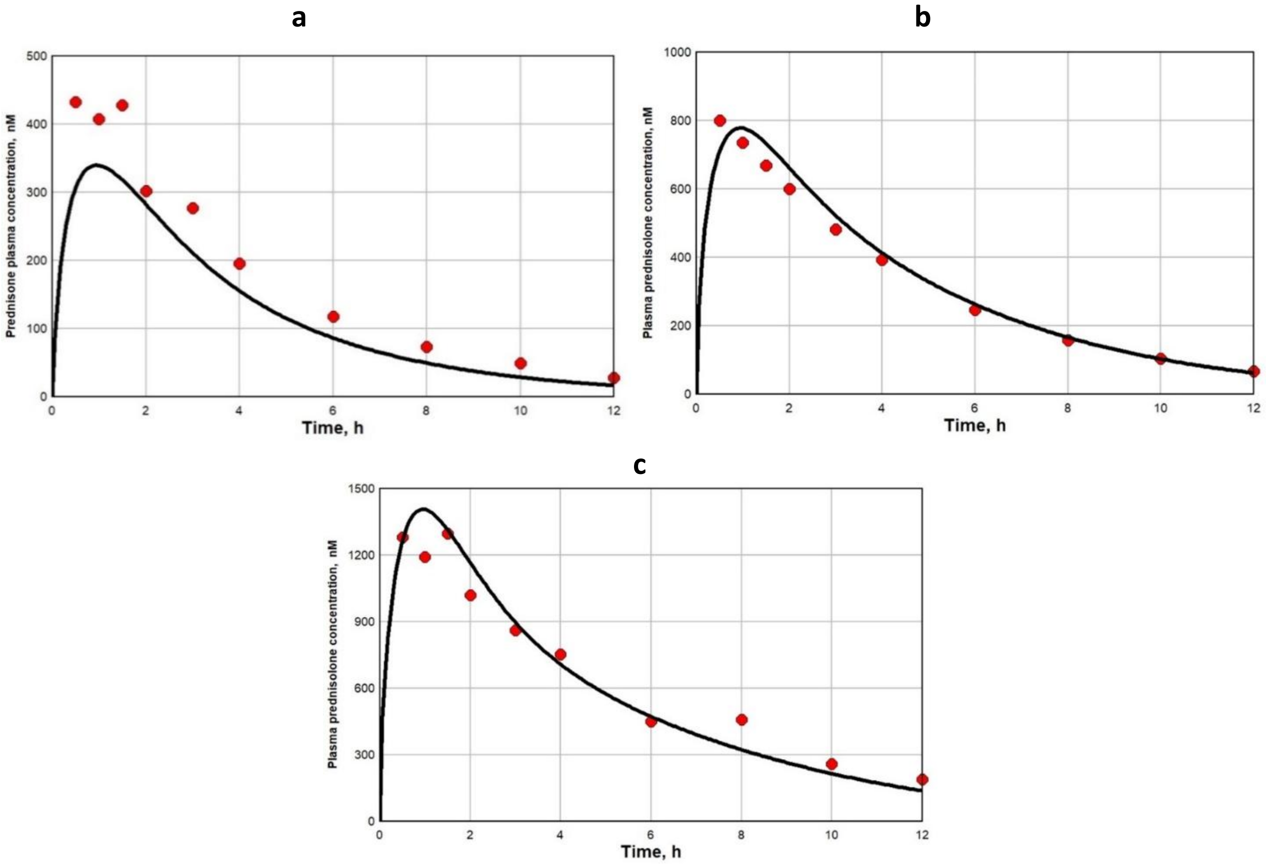

Figure S5. Comparison of the model predictions of Xu et al with the experimental results of Rose et al for the total plasma prednisolone concentrations following oral doses of 5 (a), 20 (b) or 50 (c) mg of prednisone

Summary

The purpose of this Supplementary section is to develop PK models that provide accurate predictions of the plasma glucocorticoid levels following arbitrary IV or oral doses. These plasma concentrations are then used in the main text to predict the degree of glucocorticoid mediated nuclear transcription. A summary of these PK results is provided below.

For the linear PK glucocorticoids dexamethasone and methylprednisolone, the PK are characterized by the unit response function $h(t)$ (equation (12)) whose parameters are summarized in Table S2. The plasma concentration following an arbitrary IV input ($I_{IV}(t)$), is described by equation (11):

$$C(t) = \int_0^t I_{IV}(\tau)\, h(t-\tau) d\tau \quad (25)$$

For an oral input of dose D, the plasma concentration is equal to:

$$C(t) = \int_0^t I_{GI}(\tau)\, h(t-\tau) d\tau \quad (26)$$

CC BY

where $I_{GI}(t)$ corresponds to equation (23), with the parameters *F, T_G and T_P* listed in Table S2. The binding to the glucocorticoid receptor is dependent on the free (unbound) concentration, which is simply equal to the free fraction (Table S1) times $C(t)$.

Oral prednisone is converted by the liver to the active prednisolone form, whose free concentration determines the glucocorticoid receptor binding. Because of the non-linear transcortin binding, the unit response function is not applicable, and it is necessary to use a more complicated approach. As shown above, the model described by Xu *et. al.* [16] provides a good fit to the data for the prednisone dosage range of 5 to 50 mg. The free prednisolone plasma concentration as a function of time after the oral prednisone dose is obtained as the solution to a set of three linear differential equations, characterized by 8 parameters.

References:

[1] D.G. Levitt, Computer Assisted Human Pharmacokinetics: Non-compartmental, Deconvolution, Physiologically Based, Intestinal Absorption, Non-Linear, 2017. https://www.pkquest.com/assets/docs/HumanPK3.166101304.pdf.

[2] D.G. Levitt. Quantitation of small intestinal permeability during normal human drug absorption. *BMC Pharmacology and Toxicology* **14** (2013) 34. https://doi.org/10.1186/2050-6511-14-34.

[3] M.E. Brady, G.P. Sartiano, S.L. Rosenblum, N.E. Zaglama, C.T. Bauguess. The pharmacokinetics of single high doses of dexamethasone in cancer patients. *European Journal of Clinical Pharmacology* **32** (1987) 593-596. https://doi.org/10.1007/BF02455994.

[4] P. Rohdewald, H. Mollmann, J. Barth, J. Rehder, H. Derendorf. Pharmacokinetics of dexamethasone and its phosphate ester. *Biopharmaceutics & Drug Disposition* **8** (1987) 205-212. https://doi.org/10.1002/bdd.2510080302.

[5] G.G. Toth, C. Kloosterman, D.R. Uges, M.F. Jonkman. Pharmacokinetics of high-dose oral and intravenous dexamethasone. *Therapeutic Drug Monitoring* **21** (1999) 532-535. https://doi.org/10.1097/00007691-199910000-00007.

[6] D.M. Cummings, G.E. Larijani, D.P. Conner, R.K. Ferguson, M.L. Rocci, Jr. Characterization of dexamethasone binding in normal and uremic human serum. *DICP* **24** (1990) 229-231. https://doi.org/10.1177/106002809002400301.

[7] E.A. Peets, M. Staub, S. Symchowicz. Plasma binding of betamethasone-3H, dexamethasone-3H, and cortisol-14C--a comparative study. *Biochemical Pharmacology* **18** (1969) 1655-1663. https://doi.org/10.1016/0006-2952(69)90153-1.

[8] L.E. Hare, K.C. Yeh, C.A. Ditzler, F.G. McMahon, D.E. Duggan. Bioavailability of dexamethasone. II. Dexamethasone phosphate. *Clinical Pharmacology & Therapeutics* **18** (1975) 330-337. https://doi.org/10.1002/cpt1975183330.

[9] S. Miyabo, T. Nakamura, S. Kuwazima, S. Kishida. A comparison of the bioavailability and potency of dexamethasone phosphate and sulphate in man. *European Journal of Clinical Pharmacology* **20** (1981) 277-282. https://doi.org/10.1007/BF00618778.

[10] B.T. O'Sullivan, D.J. Cutler, G.E. Hunt, C. Walters, G.F. Johnson, I.D. Caterson. Pharmacokinetics of dexamethasone and its relationship to dexamethasone suppression test outcome in depressed patients and healthy control subjects. *Biological Psychiatry* **41** (1997) 574-584. https://doi.org/10.1016/s0006-3223(96)00094-7.

[11] S.J. Szefler, W.F. Ebling, J.W. Georgitis, W.J. Jusko. Methylprednisolone versus prednisolone pharmacokinetics in relation to dose in adults. *European Journal of Clinical Pharmacology* **30** (1986) 323-329. https://doi.org/10.1007/BF00541537.

[12] S.M. Al-Habet, H.J. Rogers. Methylprednisolone pharmacokinetics after intravenous and oral administration. *British Journal of Clinical Pharmacology* **27** (1989) 285-290. https://doi.org/10.1111/j.1365-2125.1989.tb05366.x.

[13] H. Derendorf, H. Mollmann, M. Krieg, S. Tunn, C. Mollmann, J. Barth, H.J. Rothig. Pharmacodynamics of methylprednisolone phosphate after single intravenous administration to healthy volunteers. *Pharmaceutical Research* **8** (1991) 263-268. https://doi.org/10.1023/a:1015864709082.

[14] S. Rohatagi, J. Barth, H. Mollmann, G. Hochhaus, A. Soldner, C. Mollmann, H. Derendorf. Pharmacokinetics of methylprednisolone and prednisolone after single and multiple oral administration. *Journal of Clinical Pharmacology* **37** (1997) 916-925. https://doi.org/10.1002/j.1552-4604.1997.tb04266.x.

[15] J.Q. Rose, A.M. Yurchak, W.J. Jusko. Dose dependent pharmacokinetics of prednisone and prednisolone in man. *Journal of Pharmacokinetics and Biopharmaceutics* **9** (1981) 389-417. https://doi.org/10.1007/BF01060885.

[16] J. Xu, J. Winkler, H. Derendorf. A pharmacokinetic/pharmacodynamic approach to predict total prednisolone concentrations in human plasma. *Journal of Pharmacokinetics and Pharmacodynamics* **34** (2007) 355-372. https://doi.org/10.1007/s10928-007-9050-8.

CC BY
